# Preoperative Ahlbäck radiographic classification grade significantly influences clinical outcomes of double level osteotomy for osteoarthritic knees with severe varus deformity

**DOI:** 10.1186/s40634-023-00573-4

**Published:** 2023-01-25

**Authors:** Hiroshi Nakayama, Ryo Kanto, Shintaro Onishi, Kenta Amai, Ryosuke Ukon, Toshiya Tachibana, Shinichi Yoshiya, Takuya Iseki, Shota Morimoto, Tomoya Iseki

**Affiliations:** 1 Department of Orthopaedic Surgery, Hyogo Medical University, Mukogawa-Cho, Nishinomiya City, Hyogo, 653-8501 Japan; 2 Nishinomiya Kaisei Hospital, Ohama-Cho Nishinomiya City, Hyogo, 662-0957 Japan; 3 Osaka Kaisei Hospital, Miyahara Yodogawa Ward, Osaka City, Osaka, 532-0003 Japan

**Keywords:** Knee, Osteoarthritis, Double level osteotomy, Osteotomy(DLO), Ahlbäck radiographic classification

## Abstract

**Purpose:**

The purpose of this study was to examine the relationship between preoperative Ahlbäck radiographic classification grade and the clinical outcomes of double level osteotomy (DLO) performed for osteoarthritic knees with severe varus deformity.

**Methods:**

The study population comprised a consecutive series of 99 knees (68 patients) for which DLO was performed and follow-up results for a minimum of two years were available. The Ahlbäck radiographic classification system was used to determine the osteoarthritic grade. The following radiological parameters for alignment and bone geometry were measured: mechanical lateral distal femoral angle (mLDFA), mechanical medial proximal tibial angle (mMPTA), joint-line convergence angle (JLCA), and mechanical tibiofemoral angle (mTFA). Clinical results were assessed using the Knee Injury and Osteoarthritis Outcome Score (KOOS) and the International Knee Documentation Committee (IKDC) subjective score preoperatively and at 2 years after surgery. Difference between preoperative and postoperative measurements as well as relationship between Ahlbäck grade and radiological/clinical results were statistically assessed.

**Results:**

The average age of the study participants was 60.9 ± 6.2 years and the mean follow-up period was 45.4 ± 15.2 months. Each of the radiological parameters exhibited preoperative abnormal values. Knees with Ahlbäck grade 3 and 4 osteoarthritis exhibited significantly greater JLCA and mTFA than grade 1 knees. Two years post-surgery, all radiological parameter values measured within a normal range. Clinical evaluation showed significant improvement in KOOS after surgery. Analysis of the relationship between Ahlbäck grade and clinical score showed that the 2-year postoperative KOOS scores in grade 3 and 4 osteoarthritic knees were significantly lower than grade 1 knees (with the mean 2-year KOOS scores of 350.0 ± 79.9, 317.9 ± 78.3, and 420.2 ± 42.9, respectively).

**Conclusions:**

While DLO may produce significant radiological and clinical improvement in knees with joint space obliteration, Ahlbäck grade 3 and 4 osteoarthritic knees associated with larger JLCA and mTFA showed less satisfactory clinical results compared to grade 1 knees. Level of Evidence: IV case series.

## Introduction

Osteotomy around the knee is a commonly employed treatment for active patients with uni-compartmental knee osteoarthritis, and favorable outcomes have been reported [11, 16, 18]. There are several types of osteotomies available depending on the level and mode of deformity correction. A conventional osteotomy is typically performed only on the proximal tibia, however, correcting severe varus deformity with a single-level high tibial osteotomy (HTO) may result in non-anatomical joint line obliquity [1, 7, 29], which can also lead to other problems such as increased shear force on the joint [29], femoral subluxation during weight-bearing, and technical difficulty in total knee arthroplasty (TKA) conversion [1, 14, 33, 34]. In such cases, correction on both the femoral and tibial sides by double level osteotomy (DLO) have been our main surgical option with the intent of reconstructing physiological joint geometry [8, 10, 27, 29].

Several studies have examined the factors that influence the clinical outcomes of osteotomies [11, 16–18]. Among them, the severity of preoperative osteoarthritis is a factor that may affect postoperative clinical outcomes [22, 30, 39, 42]. However, the reported results of these previous studies are disparate, and the effect of preoperative osteoarthritic severity on the surgical outcomes has not been clarified. In addition, the majority of the previous studies dealt with HTO. Although some studies have reported clinical results after DLO [2, 7, 8, 28, 36], the correlation between preoperative osteoarthritic grade and DLO results has yet to be investigated.

For radiological classification of osteoarthritic severity, the Kellgren-Laurence (K-L) radiographic classification system has generally been adopted in relevant studies to date. [17, 19, 20, 30, 42]. In the K-L classification system, the grade 2 change represents osteophyte formation without joint space narrowing. Therefore, most knees with joint space narrowing that undergo osteotomy are classified as either grade 3 or 4. However, Ahlbäck proposed another radiological classification system for osteoarthritic knees in which K-L grade 4 can be further subdivided into Ahlbäck grades 2–5 [3, 41]. Since candidates for DLO present severe varus deformity with apparent joint space narrowing, use of the Ahlbäck classification may provide a clearer relationship between radiological osteoarthritic grade and surgical outcome.

Therefore, the purpose of this study was to examine the relationship between radiological severity of osteoarthritis based on Ahlbäck’s classification and radiological/clinical results after DLO in knees with severe varus deformity. It was hypothesized that severe grade in the Ahlbäck 's classification would be associated with inferior outcomes.

## Materials and Methods

### Study population and design

Patients who underwent DLO at our hospital during the study period of January 2014 to November 2019 were included in the study. The study population consisted of 68 patients (31 males and 37 females) who underwent DLO during the study period, for a total of 99 knees, all of whom could be followed-up for a minimum of 2 years after surgery. One knee was converted to a total knee arthroplasty (TKA) 3-years after DLO. The average patient age was 60.9 ± 6.2 years (range: 45 years to 75 years), and the mean follow-up period was 45.4 ± 15.2 months (range: 24 months to 89 months). During the study period, isolated OWHTO was performed on 59 knees and isolated OWDTO on 15 knees, while TCVO was performed as an isolated procedure in 15 knees and concomitantly with OWDTO in 10 knees (Fig. 2 and Table 1). Subjects were periodically followed up after surgery, and pre- and postoperative radiological and clinical data at 2 years after surgery were retrospectively reviewed for each of the knees. Those without a minimum of 2 years of complete follow-up data were excluded. Clinical results were assessed using the Knee Injury and Osteoarthritis Outcome Score (KOOS) and the International Knee Documentation Committee (IKDC) subjective score preoperatively and at 2 years after surgery. In January 2017, tibial condylar valgus osteotomy (TCVO), L-shape osteotomy in the medial tibial condyle, was introduced to our practice to reduce excessive joint-line convergence angle (JLCA) by elevating the declined medial tibial plateau [9, 21, 26]. TCVO was indicated for knees showing over a 4° JLCA on the standing radiograph and performed as an isolated procedure or concomitantly with another proximal tibial osteotomy (OWHTO or OWDTO). In August 2018, open wedge distal tuberosity tibial osteotomy (OWDTO) was introduced to our practice to avoid postoperative patella infra, which was considered an inherent complication following OWHTO. Since then, OWDTO has replaced OWHTO and OWDTO using TriS plate (Olympus, Tokyo, Japan) has been the procedure of our primary option for proximal tibial osteotomy [4, 26, 35] (Fig. 2). Design of this study corresponds to a retrospective comparative analysis of prospectively collected data at a single center with a single-surgeon setting. The contents and protocol of this study were reviewed and approved by the Review Board of the Hyogo Medical University (Approval number #2218). Indication for DLO in our practice has been described in our past reports [27]. Regarding the severity of preoperative osteoarthritis, DLO was not indicated for knees with Ahlbäck grade 5 severe osteoarthritic changes. Prior to surgery, deformity analysis and planning were performed on a weight-bearing long-leg radiograph using digital planning software (mediCAD®, Hectect, Germany) [37]. Surgical simulation for correction was initially performed using open-wedge HTO (OWHTO) alone. If the mMPTA in the simulation of isolated OWHTO was 95° or greater (or the wedge size was 15 mm or greater), then a correction on both the femoral and tibial side by DLO was considered as a surgical option, and surgical planning was conducted accordingly. The intended radiological parameters for the simulated surgery were set at 85° for mLDFA and 90° for the mMPTA. The mechanical tibiofemoral angle (mTFA) was aimed at 0.5°-1° (a slight valgus position) from 2014 to 2017 [27], but changed to 2° in 2018.

**Table 1:** HTO classification and proportion

Ahlbäck grade	Total (knees)	OWHTO (knees)	OWDTO (knees)	TCVO (knees)	TCVO/OWDTO (knees)
1	11	7	2	1	1
2	41	27	10	2	2
3	42	22	2	11	7
4	5	3	1	1	0
total	99	59	15	15	10

### Surgical procedure

All surgeries were performed by the first author (H.N.), and a detailed account of the surgical procedure was described in our previous paper [27]. Arthroscopic examination was performed prior to the osteotomy, and intraarticular arthroscopic procedures such as meniscal surgeries were performed when deemed necessary. The DLO began with a closed-wedge distal femoral osteotomy (LCW-DFO). A 4–5 cm longitudinal incision was made on the lateral side of the femur just above the femoral epicondyle, and osteotomy was performed using the biplanar technique according to the MIPO procedure [12, 43]. The TomoFix MDF anatomical plate (DePuy Synthes, Solothurn, Switzerland) originally designed for the contralateral side was used for fixation and bent according to the individual’s anatomy. In May 2018, the fixation device was changed to a TriS LDFO plate (Olympus, Tokyo, Japan) [25]. After LCW-DFO, osteotomy was completed with OWHTO. The OWHTO was performed following a modified technique reported by Staubli and Lobenhoffer [23, 40]. A bone substitute (ß-TCP: Osferion 60; Olympus Terumo Biomaterials, Tokyo, Japan) was inserted into the osteotomy gap. Fixation was achieved using a TomoFix TM medial high tibial plate (DePuy Synthes, Solothurn, Switzerland). In January 2017, tibial condylar valgus osteotomy (TCVO), L-shape osteotomy in the medial tibial condyle, was introduced to our practice to reduce excessive joint-line convergence angle (JLCA) by elevating the declined medial tibial plateau [9, 21, 26]. TCVO was indicated for knees showing over a 4° JLCA on the standing radiograph and performed as an isolated procedure or concomitantly with another proximal tibial osteotomy (OWHTO or OWDTO). In August 2018, open wedge distal tuberosity tibial osteotomy (OWDTO) was introduced to our practice to avoid postoperative patella infra, which was considered an inherent complication following OWHTO. Since then, OWDTO has replaced OWHTO and OWDTO using TriS plate (Olympus, Tokyo, Japan) has been the procedure of our primary option for proximal tibial osteotomy [4, 26, 35] (Fig. 2).

### Postoperative rehabilitation

The operated knee was not immobilized, and range of motion exercises began the day after surgery without any restriction. Patients were partial weight-bearing three weeks post-surgery, using crutches and 20 kg of body weight. By week 4, patients were fully weight bearing [27, 38].

### Radiological evaluation

**Fig. 1:**
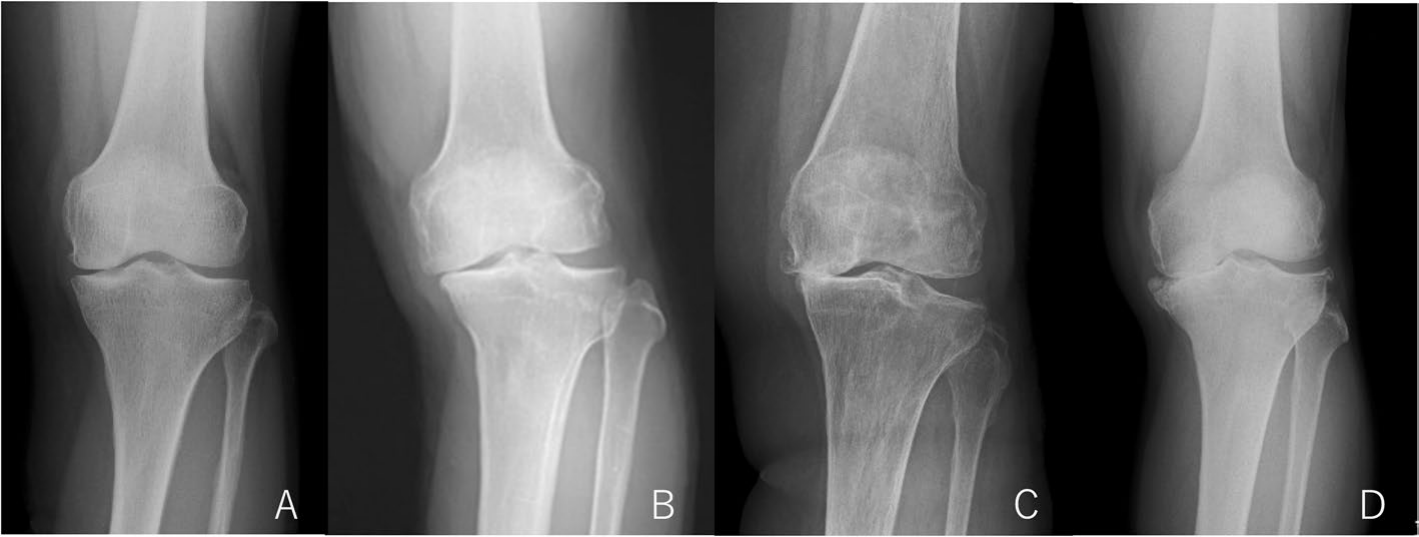
Ahlbäck radiographic classification grade

During radiological evaluation, the severity of osteoarthritis was assessed by a weight-bearing radiograph according to the Ahlbäck classification system: grade 1, reduction of joint space < 3 mm; grade 2, joint space obliteration; grade 3, minor tibial plateau attrition < 5 mm; grade 4, moderate attrition of 5-10 mm; and grade 5, severe attrition > 10 mm with anterior tibial subluxation [3] (Fig. 1). As for the radiological parameters for alignment and bone/joint geometry, the following angles were measured on a long-leg weight-bearing radiograph using dedicated software (mediCAD): mechanical lateral distal femoral angle (mLDFA), mechanical medial proximal tibial angle (mMPTA), joint-line convergence angle (JLCA), and mechanical tibiofemoral angle (mTFA) [27, 38]. The excellent measurement reliability of this analytical imaging software was confirmed in the previous study [37].

### Clinical evaluation

Clinical outcomes were assessed using the patient-reported outcome measures (PROMs) preoperatively and at the periodical follow-up for each of the knees. For the assessment, pre- and 2-year postoperative Knee Injury and Osteoarthritis Outcome Score (KOOS) and International Knee Documentation Committee (IKDC) subjective scores were obtained and subjected to the analysis.

### Statistical analysis

Comparison of pre- and postoperative results was performed using the Mann–Whitney U test. The relationship between the Ahlbäck classification grade and clinical and radiological parameters was statistically evaluated using the one-way ANOVA and Tukey’s post hoc test. The statistical significance level was set at P < 0.05. As for the power analysis, a priori power calculation was not feasible due to the lack of relevant data in prior studies. A post-hoc power analysis was conducted to assess the statistical power with the sample size of the present study. A free statistical power analysis software (G*Power, version 3.1.9.2; Franz Faul, Universitat Kiel) was used for the calculation. Consequently, it was calculated that the total sample size in this study could achieve a statistical power of 0.74 for pre- and postoperative comparison and 0.99 for comparison among 4 groups at α = 0.05 and effect size = 0.5.

## Results

### Radiological results

Preoperatively, the mean mTFA angle was -13.6 ± 2.9° (varus) (range; -8.8° to -23.6°), the mean mLDFA was 90.6 ± 1.9° (range; 88.8° to 95°), and the mean mMPTA was 82.1 ± 2.1° (range; 77.2° to 86.4°), indicating a varus malalignment consisting of combined femoral and tibial deformities. In addition, preoperative JLCA averaged 5.4 ± 2.6° (range; 2.5° to 10.8°), showing an intraarticular component of varus deformity associated with increased ligament laxity. Two-years post-surgery, all radiological parameters were corrected to normal range values (Table 2).

**Table 2:** Pre- and postoperative radiological parameter values for alignment and geometry of the knee

	Preoperative measurement ( °)	Postoperative 2-year measurement ( °)
mTFA	13.6 ± 2.9 varus	0.0 ± 3.7
mLDFA	90.6 ± 1.9	86.0 ± 1.7
mMPTA	82.1 ± 2.1	89.2 ± 2.5
JLCA	5.4 ± 2.6	3.5 ± 2.4

mLDFA: mechanical lateral distal femoral angle

mMPTA: mechanical medial proximal angle

mTFA: mechanical tibio-femoral angle

JLCA: joint-line convergence angle

According to Ahlbäck radiographic classification, 11 knees (11.1%) were grade 1, 40 knees (40.4%) were grade 2, 43 knees (43.5%) were grade 3, and 5 knees (5.0%) were classified as grade 4 for preoperative osteoarthritic severity. Analysis of the relationship between preoperative Ahlbäck grade and radiological parameters showed significant differences in preoperative JLCA values between group 1 and groups 3 and 4 (*P* < 0.01), and preoperative mTFA values between grade 1 and grade 2, 3 and 4 knees (*P* < 0.01) (Table 3).

**Table 3:** Relationships between preoperative knee parameters and Ahlbäck grade

Ahlbäck grade	Preoperative mLDFA ( °)	Preoperative mMPTA ( °)	Preoperative mFTA ( °)	Preoperative JLCA ( °)
1	90.9 ± 1.3	81.7 ± 1.8	11.0 ± 1.8 varus	2.1 ± 1.6
2	90.8 ± 1.8	81.8 ± 2.4	12.8 ± 2.7 varus	4.0 ± 1.7 ^†^
3	90.5 ± 2.1	82.4 ± 2.0	14.7 ± 2.6 varus ^*^	6.9 ± 1.7 ^†^
4	89.5 ± 0.4	82.7 ± 0.5	16.2 ± 1.5 varus ^*^	9.3 ± 1.4 ^†^

Significant differences in mTFA between grade 1 and grade 3, 4 knees by one-way ANOVA with Turkey’s post hoc test (*P < 0.01)

Significant differences in JLCA between grade 1 and grade 2, 3, 4 knees by one-way ANOVA with Turkey’s post hoc test (^†^P < 0.01)

mLDFA: mechanical lateral distal femoral angle

mMPTA: mechanical medial proximal angle

mTFA: mechanical tibio-femoral angle

JLCA: joint-line convergence angle

Clinical results.

Preoperatively, the mean KOOS and IKDC subjective scores were 186.6 ± 71.9 and 32.8 ± 14.1, respectively. One- and two-years post-surgery, these scores increased to 351.8 ± 75.3 and 374.1 ± 76.9 for the KOOS and 61.9 ± 13.6 and 68.2 ± 14.6 for the IKDC subjective scores, showing significant postoperative improvement in both PROMs (Table 4).

**Table 4:** Clinical scores based on patient-reported outcome measures

Scores	Preoperative	Postoperative 2-year
KOOS (Points)	186.6 ± 71.9	374.1 ± 76.9^*^
IKDC (Points)	32.8 ± 14.1	68.2 ± 14.6^*^

^*^Statistically significant improvement compared to preoperative scores (^*^P < 0.05)

KOOS: Knee Injury and Osteoarthritis Outcome Score

IKDC: International Knee Documentation Committee

With regard to the relationship between the PROMs and Ahlbäck classification, the mean preoperative KOOS scores were 185.2 ± 46.6 for grade 1, 182.0 ± 70.4 for grade 2, 192.5 ± 79.1 for grade 3, and 171.7 ± 38.2 for grade 4, showing no significant differences between the KOOS score and the Ahlbäck grade. At the 2-year follow-up evaluation, the mean KOOS scores were recorded as 420.2 ± 42.9 for grade 1, 393.9 ± 67.3 for grade 2, 350.0 ± 79.9 for grade 3, and 317.9 ± 78.3 for grade 4, each having shown significant improvement regardless of Ahlbäck grade (*P* < 0.01). Statistical analysis of the effect of preoperative Ahlbäck grade on the postoperative clinical outcomes showed that the 2-year KOOS scores for the knees with grade 3 and 4 osteoarthritis were significantly lower than that in grade 1 knees (*P* = 0.027 and 0.003, respectively) and the score in grade 4 knees was significantly lower than that in grade 2 knees (*P* = 0.027) (Table 5). Regarding the analytical results for the KOOS sub-scores, no significant relationship was demonstrated between KOOS sub-scores and Ahlbäck 's classification grade (Table 6). As regards the IKDC score, there was no significant difference in the postoperative IKDC score between the groups with different preoperative Ahlbäck grade (Table 7). In order to assess the statistical power of this study, a post hoc power analysis was conducted for comparison of the pre-and postoperative KOOS and IKDC subjective scores using Mann–Whitney U test and comparison among the 4 groups using ANOVA. Consequently, it was calculated that the total sample size in this study could achieve a statistical power of 0.74 for pre- and postoperative comparison and 0.99 for comparison among 4 groups at α = 0.05 and effect size = 0.5.

**Table 5:** Relationship between KOOS and Ahlbäck grade

Ahlbäck grade	Preoperative KOOS	Postoperative 2-year KOOS
1	185.2 ± 46.6	420.2 ± 42.9
2	182.0 ± 70.4	393.9 ± 67.3
3	192.5 ± 79.1	350.0 ± 79.9^*^
4	171.7 ± 38.2	317.9 ± 78.3^*†^

Significant differences in postoperative 2-year KOOS between Ahlbäck grade 1 and grade 3, 4　(* *P* < 0.01)　and between Ahlbäck grade 2 and grade 4　(^†^ *P* < 0.01) by one-way ANOVA Post-hoc Tukey’s test

*KOOS*: Knee Injury and Osteoarthritis Outcome Score

**Table 6:** Relationship between KOOS sub-score and Ahlbäck grade

Ahlbäck grade	KOOS domains	Preoperative KOOS	Postoperative 2-year KOOS
1	Symptoms	45.6 ± 13.0	85.4 ± 7.8
	Pain	40.1 ± 19.9	90.3 ± 6.7
	ADL	58.3 ± 22.3	93.8 ± 6.1
	Sports	18.9 ± 13.1	74.0 ± 14.6
	QOL	22.2 ± 11.5	78.8 ± 20.6
	Total	185.2 ± 46.6	420.2 ± 42.9
2	Symptoms	40.8 ± 18.0	79.9 ± 14.1
	Pain	41.0 ± 18.6	87.4 ± 9.7
	ADL	55.1 ± 17.2	89.6 ± 9.6
	Sports	16.5 ± 16.5	64.7 ± 24.4
	QOL	28.5 ± 16.4	72.0 ± 21.4
	Total	182.0 ± 70.4	393.9 ± 67.3
3	Symptoms	45.8 ± 21.2	76.6 ± 16.8
	Pain	43. 3 ± 20.0	79.0 ± 18.0
	ADL	56. 3 ± 18.5	84.1 ± 14.1
	Sports	19.0 ± 17.4	50.8 ± 28.4
	QOL	28.0 ± 16.8	63.1 ± 24.2
	Total	192.5 ± 79.1	350.0 ± 79.9^*^
4	Symptoms	47.3 ± 9.2	78.6 ± 14.6
	Pain	31.2 ± 18.3	78.7 ± 7.0
	ADL	45.6 ± 11.2	90.2 ± 5.5
	Sports	22.5 ± 10.3	68.3 ± 9.4
	QOL Total	25.0 ± 11.7 171.7 ± 38.2	81.2 ± 13.5 317.9 ± 78.3^*†^

Significant differences in postoperative 2-year KOOS between Ahlbäck grade 1 and grade 3, 4　(* *P* < 0.01)　and between Ahlbäck grade 2 and grade 4　(^†^ *P* < 0.01) by one-way ANOVA Post-hoc Turkey’s test

*KOOS:* Knee Injury and Osteoarthritis Outcome Score

**Table 7:** Relationship between IKDC subjective score and Ahlbäck grade

Ahlbäck grade	Preoperative IKDC	Postoperative 2-year IKDC
1	36.0 ± 12.4	70.9 ± 16.5
2	30.3 ± 13.0	70.5 ± 11.5
3	32.3 ± 14.5	63.5 ± 15.5
4	22.7 ± 14.0	62.6 ± 16.8

No significant differences in postoperative 2-year IKDC between Ahlbäck grades

*IKDC:* International Knee Documentation Committee

## Discussion

**Fig. 2:**
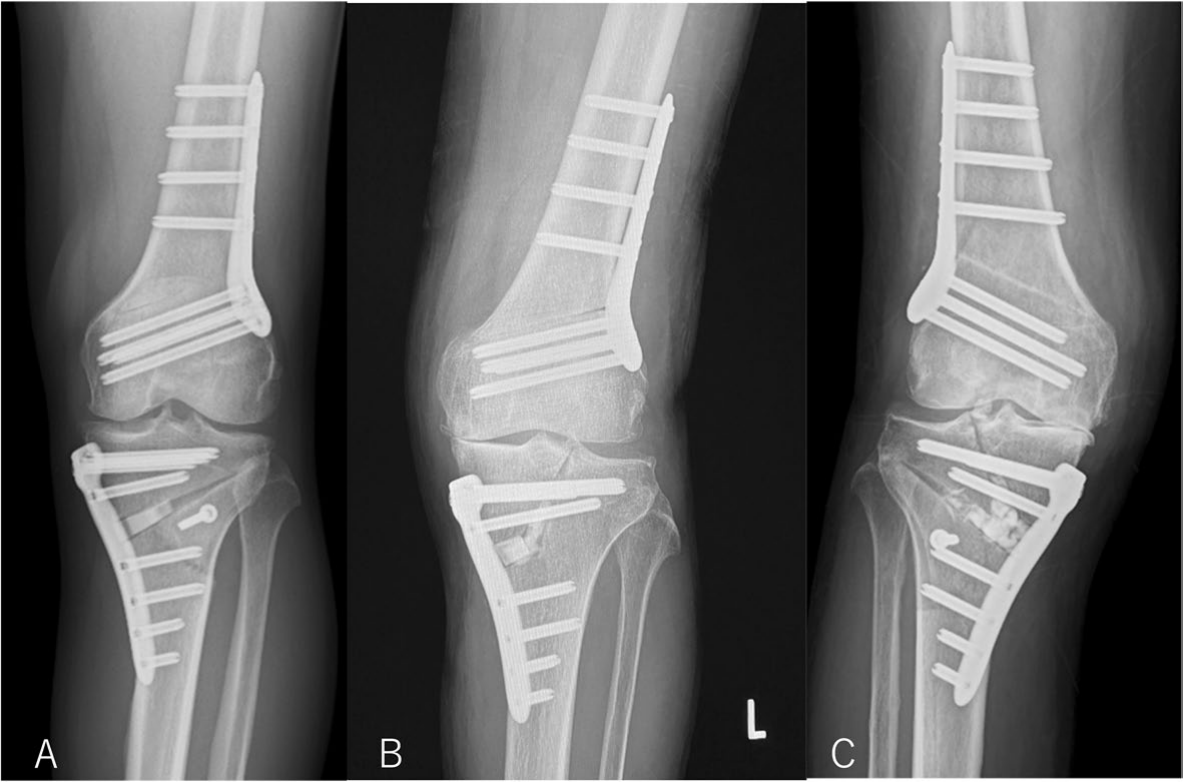
Postoperative weight-bearing anteroposterior radiographs after DLO, A: LCW-DFO + OWDTO (left knee), B: LCW-DFO + TCVO (left knee), C: LCW-DFO + TCVO + OWDTO (right knee), DLO: double level osteotomy, LCW-DFO: lateral closed-wedge distal femoral osteotomy, TCVO: tibial condylar valgus osteotomy, OWDTO: open wedge distal tuberosity osteotomy

The most important finding was that the KOOS results in Ahlbäck grades 3 and 4 knees were significantly inferior compared to those in grade 1 at 2 years after surgery. In addition, radiological analyses showed that joint line convergence (JLCA) and varus deformity (mTFA) were significantly greater in knees with advanced Ahlbäck osteoarthritic classification grade. These study results show that severe osteoarthritic changes in the Ahlbäck classification associated with advanced bone/joint deformity can be a prognostic factor predicting less satisfactory clinical outcomes. In previous studies dealing with single level HTO, severe knee osteoarthritis equivalent to K-L grade 4 was considered a contraindication or a poor prognostic cohort with poor surgical results. In our practice, DLO has been indicated for knees associated with both tibial and femoral deformities to avoid joint-line obliquity as a sequel to single-level correction on the tibial side only [7, 27, 29, 38]. Anatomical correction of the bone and articular geometrical parameters results in the mechanical axis of the leg passing through and around the center of the articular surface of the knee [6, 27, 31, 32, 38]. The majority (89%) of the knees in this study presented grade 4 K-L osteoarthritic changes. In contrast to the previous studies reporting unfavorable outcome of isolated HTO performed for this group of knees [30, 39, 42], the present study showed that DLO could yield significant clinical/radiological improvements even for advanced osteoarthritic knees. Consequently, the results of this study support the efficacy and validity of our surgical strategy of anatomical reconstruction of the bone and joint geometry via DLO [5, 7, 15, 27, 38]. In recent years, the surgical procedure for DLO has evolved to incorporate biplanar osteotomy and minimally invasive plate osteoosynthesis (MIPO) techniques [10, 27, 38], and the results have been reported in several studies [10, 27, 38]. However, no studies have analyzed the clinical results according to osteoarthritic grade. In this study, we compared the outcomes of DLO for different Ahlbäck grades. Previous studies have widely used the K-L grading system to determine the severity of osteoarthritis, but since most subjects who underwent DLO presented K-L grade 4 changes in this study, the use of the Ahlbäck classification system was able to more effectively differentiate the osteoarthritic severity. In addition, the Ahlbäck classification was better suited for radiological evaluation of this patient population than the K-L classification because it focuses more on joint space narrowing and bone attrition [41]. In the statistical analysis of this study, improvement in clinical score was less satisfactory in knees with Ahlbäck grade 3 or 4. Radiological assessment showed a significantly increased JLCA and severe varus deformity (mTFA) associated with attrition of the medial tibial plateau especially in Ahlbäck grade 3 or 4 knees. In such cases, osteotomy may not achieve reconstruction of physiological bone and joint anatomy. There was an illustrative case in this series showing clinical failure after DLO. This case exhibited large JLCA (9.7°) during preoperative evaluation. Postoperatively, the JLCA was 8.3° with a postoperative mTFA of 5.5° varus indicating a recurrent (uncorrected) deformity (Fig. 2). The operated knee was eventually converted to TKA two years after DLO, therefore, was considered as failure. Based on our clinical experiences with DLO performed for Ahlbäck grade 3 or 4 knees, modification of our surgical strategies in the aspects of procedure or indication seems required for this group of knees.

Increased JLCA equates to intra-articular deformity [24], and has been reported to worsen long-term clinical outcomes after HTO [13, 20, 24]. In this situation, concomitant TCVO may be able to reduce the JLCA [9, 21]. Considering this issue, our surgical strategy for varus knees with a large JLCA angle was changed to concomitant TCVO with DLO from 2017 [9, 21, 35].

**Fig. 3:**
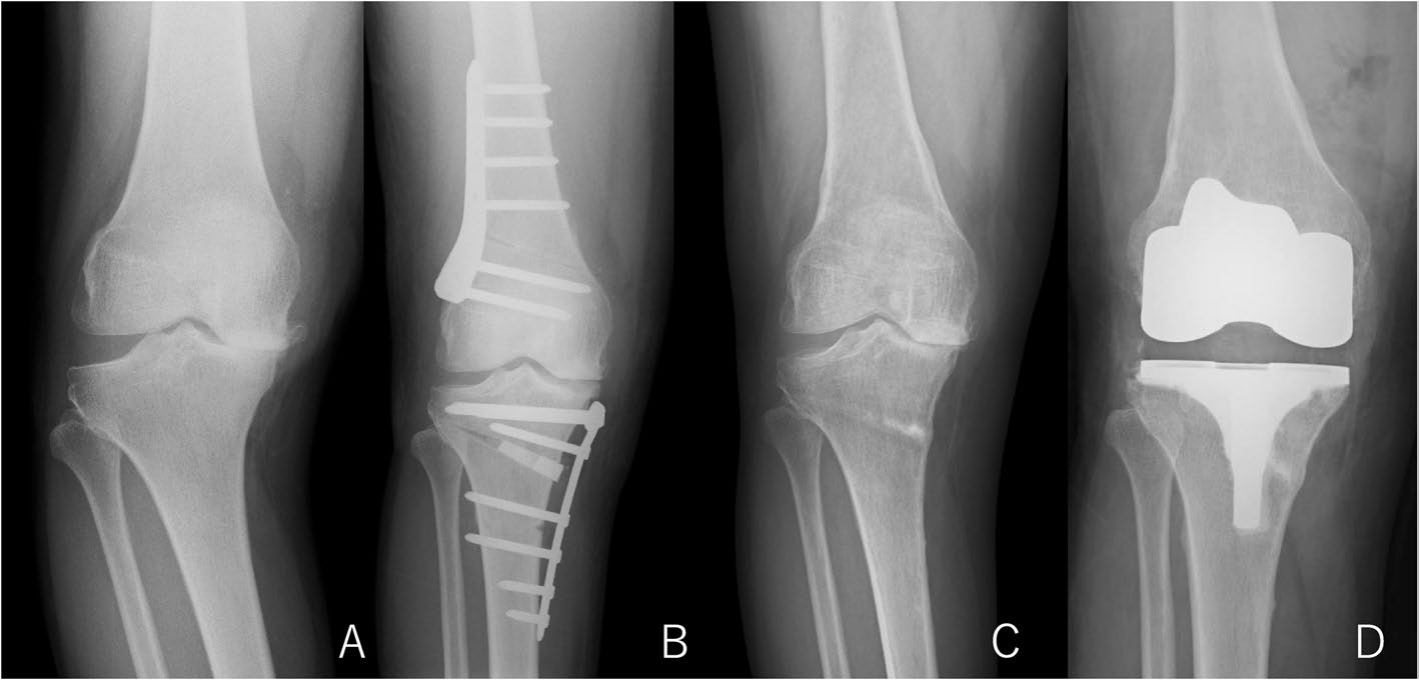
Serial radiographs of the right knee converted to TKA following DLO, A: A weight-bearing anteroposterior radiograph preoperative JLCA is large (9.7°). B: An anteroposterior radiograph following DLO. C: A weight-bearing anteroposterior radiograph 2 years after DLO. Varus alignment was found to be associated with recurrence of large JLCA. D: An anteroposterior radiograph following TKA conversion, JLCA: joint line convergence angle, DLO: double level osteotomy, TKA: total knee arthroplasty

Considering the indications for DLO, the review of our clinical experiences in this study shows that clinical results after osteotomy around the knee may not be satisfactory in all knees. Some papers have reported that the preoperative degree of OA and cartilage damage can be significant risk factors for less satisfactory outcomes following HTO [13, 20, 39, 42]. Like HTO, DLO was shown to have potentially unsatisfactory results in knees with advanced OA stages corresponding to Ahlbäck grade 3 or higher. DLO also has the disadvantages of a longer postoperative recovery period and the need for hardware removal compared to TKA. Surgeons should consider these issues when determining the surgical options for knees with osteoarthritic changes of Ahlbäck grade 3 or higher (Fig 3).

There are several limitations included in this study. First, the follow-up period was short (at least 2 years), and the number of included subjects was small. Second, the study design was a retrospective analysis of a case series, and only patient-reported outcome measurements (IKDC and KOOS) were evaluated in the clinical assessment. Third, over the course of the study period, osteotomy procedure on the tibial side has been changed in our practice by introduction of TCVO for knees with large JLCA in 2017 and adoption of OWHTO replacing OWDTO in 2018. The inclusions of the cases undergoing multiple surgical options on the tibial side added to the confounding variables in the data analysis. Moreover, 31 of the 68 patients underwent DLO in both knees, and cases with both bilateral and unilateral surgeries were included in the analysis. This issue posed another source of heterogeneity in the patient profile.

Fourth, the alignment goal for DLO in this series was set to almost neutral, while the optimal alignment is yet to be defined. Fifth, radiographic measurements were performed by a single observer, and inter- and intra-observer reliabilities in the measurement of radiological parameters were not examined. However, the high intra- and interrater reliability of the digital planning software used in this study was confirmed in a previous study [37]. Finally, during the study period, our practice introduced the use of concomitant TCVO for knees with large JLCA in 2017, and OWHTO was replaced by OWDTO in 2018. However, subjects who underwent the different procedures were combined and included in the analysis of this study.

## Conclusions

While double level osteotomy may produce significant radiological and clinical improvement in knees with joint space obliteration, Ahlbäck grade 3 and 4 osteoarthritic knees associated with larger joint-line convergence angle and mechanical tibiofemoral angle showed less satisfactory clinical results compared to grade 1 knees.
